# Friston, Free Energy, and Psychoanalytic Psychotherapy

**DOI:** 10.3390/e26040343

**Published:** 2024-04-18

**Authors:** Jeremy Holmes

**Affiliations:** Department of Psychology, University of Exeter, Exeter EX4 4QG, UK; j.a.holmes@btinternet.com

**Keywords:** free energy, Friston, psychoanalysis, Winnicott

## Abstract

This paper outlines the ways in which Karl Friston’s work illuminates the everyday practice of psychotherapists. These include (a) how the strategic ambiguity of the therapist’s stance brings, via ‘transference’, clients’ priors to light; (b) how the unstructured and negative capability of the therapy session reduces the salience of priors, enabling new top-down models to be forged; (c) how fostering self-reflection provides an additional step in the free energy minimization hierarchy; and (d) how Friston and Frith’s ‘duets for one’ can be conceptualized as a relational zone in which collaborative free energy minimization takes place without sacrificing complexity.

## 1. Introduction

We know that psychoanalytic psychotherapy ‘works’, i.e. meets rigorous standards of effectiveness and efficacy [1,2,3] comparable to other forms of therapy, such as cognitive–behavioural therapy (CBT). What remains thus far unelucidated is *how* it works. There is convincing evidence that ‘common factors’, especially the therapeutic alliance, predict good outcomes, whatever the declared modality of therapy [4]. In pursuit of this problem, the free energy principle (FEP) and active inference have been brought to bear on CBT [5] and a less well-known therapeutic modality, coherence therapy [6]. Others [7,8,9,10] have considered FEP in relation to psychoanalytic work. This speculative non-systematic review is a non-mathematical and possibly naive attempt to show how Karl Friston’s ideas can help unravel the thorny issue of psychoanalytic psychotherapy’s ‘mechanisms of action’.

## 2. Methods

This discussion builds on two facets of Friston’s oeuvre. The first is the *free energy principle* (FEP) [11,12]. The second is his concept of ‘*duets for one*’ [13]. The latter, I shall argue, resonates with the psychoanalyst Donald Winnicott’s concept of *transitional space* which can, in the Fristonian model, be conceptualised as the locus for higher levels of relational free energy minimizing.

### 2.1. Radical Uncertainty

The FEP starts from the observation that, at a fundamental biological level, we live in a world of *radical uncertainty*. In common with all living entities, our knowledge of ourselves, of others, and of what is to come is inescapably constrained. We are limited by the *veil of ignorance* drawn by the narrow range and unavoidable ‘noise’ of our senses; by the fact that the brain, Markov-wise, is only statistically connected with the surrounding ‘world’ and therefore relies on inference rather than direct observation about its state(s); by time’s unidirectional arrow; and by the tendency towards entropy, which, according to the second law of thermodynamics, is where ‘we’—ourselves, our species, our planet, and our universe—are in both the short and long run heading.

### 2.2. Negentropy

In the Fristonian model, we negotiate these uncertainties in two main ways: (a) by making *Bayesian predictions*, based on prior experience and current sensory information about the likely state of the world; and (b) by *active inference*, in which we test these predicted outcomes via action/agency. This Bayesianism is embedded in the wider context of the need to stave off entropy. In order to survive and thrive, an organism must minimise the disruptive forces with which it is beset. Staying alive, both as whole organisms and at a biomolecular level, depends on order and the maintenance of boundaries, the very antithesis of entropic disorder. Thus, as quantum physicist Erwin Schrödinger [14], asking himself ‘*What is life?*’ succinctly put it, life is ‘negentropy’.

For motile organisms, this is where nervous systems and their brains come in. Brains are ‘allostats’, i.e., systems that remain stable by being able to vary according to environmental conditions [15]. The brain monitors, integrates, and regulates incoming information about the state of the world and the inner condition of the organism, while driving the muscular movements needed to increase the precision of and responses to this information. Thus, the respiratory centre in the medulla monitors oxygen levels in the blood, drives heartbeat and respiratory rates up or down according to its register, and, when necessary, motivates the animal to move to a less oxygen depleted environment. 

In the Friston model, allostasis applies not just to the whole organism but to the brain itself. The brain’s aim, as best it can, is to *minimise free energy*. Free energy, or energy unbound, refers to deviations from expected levels—in the above case, oxygen saturation. One of Friston’s many smart moves was to suggest that the energy that matters most to the brain is informational energy, and that ‘bound’ energy is information that correlates with prior expectation and so denotes order, while free energy—‘unbound’ and for which there is no corresponding top-down model or ‘prior’—equates to entropy and is therefore disruptive. 

The idea of top-down free energy minimization (FEM), like heliocentrism, is counter-intuitive. As Wittgenstein put it, why would one *not* think that the sun went round the earth. Similarly, *pace* Isherwood [16], it is surprising to think that we are *not* cameras, reproducing a pristine vision of ‘the world’ derived from the senses and registered or represented in the cerebral cortex. But, in the Fristonian model, we see not with our eyes but with our minds. The eye responds preferentially to light/dark boundaries, and so, even at the peripheral level, it is imposing top-down affordances on sensation, given that such boundaries are likely to be salient to an organism’s survival [17]. Our visions and versions of the world are more akin to testable illusions than mirrored reproductions [18]. 

Based on prior experience, we build up a model of ‘our’ world and its affordances [19], or *Regimes of Expectations* [20]—that is, those features which *matter* to us, for survival, security, pleasure, and reproduction. Via our brains, we continuously compare and correlate our priors, i.e., ‘top-down’ working models of the world, with incoming information, derived ‘bottom-up’ from our senses, external and internal. Where there is correspondence, the incoming sensory information/energy is ‘bound’ and can be taken for granted. Where there are decisions to be made, we ‘out-source’ FEM via deontic cues from the environment, telling us ‘what to do’ (e.g., stop at red traffic lights), thereby relieving the brain from working out every daily decision ab initio. 

Attention is alerted by and focuses on the residual free energy left over from top-down/bottom-up discrepancies/anomalies. These in turn trigger *active inference*, leading to minor or major readjustments of our preformed models of ‘reality’. Perhaps Leonardo’s *Mona Lisa* continues to excite admiration and wonder half a millennium after it was painted because of the ambiguity of its enigmatic and so attention-grabbing ‘smile’—invitation, coy rejection, smug satisfaction, mild misery, or merely impenetrable mystery?

### 2.3. Hierarchies

From the brain’s point of view (to adopt a theory of mind of theory of mind), free energy arises out of the discrepancies between its models of the world and the incoming information provided by the senses. Surprisal, or Shannon information, is a measure of the probability of an event. The less expected an event is, the higher its surprisal value. Friston suggests that this probabilistic process occurs hierarchically within the brain, from sense organs to the higher reaches of consciousness. 

At each stage in the ascending hierarchy, residual discrepancies between incoming sensory information and preformed models are passed on upwards for further minimisation. Eventually (we are talking milliseconds here), unbound free energy reaches the prefrontal cortex and, presumptively at least, consciousness, including *thinking about thinking*. Active inference ensures that this goes on continuously and dynamically in an inference/action loop comprising sensation, free energy minimisation, and action. 

### 2.4. Active Inference

Active inference ensures that we adaptively align ourselves and our top-down models with the state of play of the world—both inner and outer—as we perceive it. ‘Alignment’ here entails addressing ‘prediction error’—the discrepancy between prediction and sensory information. Prediction error is reduced by changing either the prediction (through perception), the evidence (through action), or the precision (through attention). Among other possible actions, we enhance the *precision of perceptions* by looking more closely, for example, at *La Giaconda*. On the basis of this, we *generate modified models* that correspond with the world as it appears. We might conclude that the *Mona Lisa*’s ambiguity is not there to be deconstructed but is precisely what its painter intended. Here, surprisal is embraced and used to stimulate *creative adaptation*. 

Another strategy that is often seen in the psychopathology which is the concern of psychotherapists, is to *move into and create environments* where the contours of reality are constrained in ways that correspond with our pre-existing models. We create the very ‘world’—or a deformed anachronistic version of it—which we expect. In psychotherapy settings, this is construed in terms of ‘transference’. Here, the therapist is erroneously seen, or constrained to behave, in ways based on the client’s preformed assumptions, i.e., in terms of past attachment figures, rather than corresponding with the reality of the present moment. The reticence and anonymity of the therapist deliberately creates an ambiguous stimulus which enables these priors to come more clearly into view. 

To illustrate this, let us return to the hierarchical nature of bottom-up/top-down surprise minimisation. As information ascends the minimisation ladder, specificity increases: round object/mouth-and-eyes/human/male/mid-fifties/in familiar attire/recognizable gait—this can be no one other but my brother. At each step along the way, *surplus uncertainty* is passed up to the next level, where the relevant repertoire of top-down models is deployed until a statistical best fit is found. It is only at this late-stage point that consciousness supervenes.

### 2.5. Wish Fulfilment

Here, one can add another layer, often unconscious, that is of vital relevance to psychotherapy. Because the process is probabilistic, allowance must be made for error. After all, my perception—although perhaps better thought of as a *perceptive construction*—might be wrong. Perhaps that is just someone who *looks like* my brother. And, what is more, maybe I just *wanted* him to turn out to be my brother, because I have not seen him for ages. In this sense, unconscious perception is motivational, ‘wish-fulfilling’, or reward-driven—in broad daylight, not just in our dreams. But evolutionary success and, arguably, ‘happiness’ ultimately depend on being in touch with reality, with things as they are rather than as we would wish them to be. We need our dreams, but only if they are realizable. The Markov blanket is where this wishfulness meets ‘reality’ in the form of sensory input. In this sense, as Friston and colleagues perceptively put it, ‘all thinking is wishful thinking’ [21]. But if wish/reality discrepancy is too great, or the capacity for top-down adjustment is impaired, where wish and fact just do not match, psychopathology can result.

To overcome this problem, social species such as ours employ a further layer in which the very assumptions deployed are questioned, and the possibilities of wish-mediated error and the inescapable ‘noise’ of a subject-to-error sense organ are taken into account. ‘That might be my brother—how I wish it was—but, equally, it could be his double; let’s get more information’—actively infer—to minimise error and resolve the question’. In neuroscientific terms [8,22], my surprise-minimisation apparatus needs to include a self-referential or ‘mentalizing’ aspect which takes my wishes and how they shape my perceptions into account. These will in turn stimulate further error-minimising strategies—for example, taking a closer look at the putative brother, and, as we shall see, asking my *companion* if she/he recognises that person.

Thus far then, the Fristonian model illuminates a number of psychotherapeutic techniques and manoeuvres. By presenting a somewhat ambiguous stimulus, the neutrality and reticence of the therapist disrupts automatic bottom-up/top-down correlation and so brings to light the client’s anachronistic priors. Mobilising the client’s agency in so doing stimulates the processes of active inference, often in abeyance in the defeated and depressed. Becoming conscious of these unconscious processes adds another layer to the hierarchical surprise-minimising millefeuille. 

This thinking about thinking gives subjects a greater understanding of their world and actions, thus in turn enhancing their likelihood of more skilful living. Learning to be self-reflective takes account of and counteracts the built-in error and limitations of our perceptions of the world [23,24]. Generating more subtle and complex top-down models of themselves and their world enables clients to live in a more adapted and fulfilling way. 

But how does psychotherapy bring about these changes? Here, we draw on a further Fristonian concept which overlaps with a more traditional psychoanalytic framework: ‘duets-for-one’, or, in terms of this paper, *relational* FEM.

## 3. Discussion

### 3.1. Negative Capability

The kernel of my argument is that, in any real-life ‘present moment’, there is always a quantum of *unminimised free energy* with which the mind/brain has to contend—and/or creatively develop—but which remains beyond the reach of non-conscious minimisation manoeuvres. The world as it exists and our knowledge of the world are never fully in kilter. This area of *necessary uncertainty* is a form of life in a living brain, but life that cannot on its own readily or fully be formulated, represented, or regulated. Since the postulated aim of the FEP is to reduce surprisal (the upper bound of which is variational free energy), the subject reduces error in two ways: (a) internally by reducing sensory noise and (b) externally by ’foraging’ the environment for ‘truthfulness’ of the sensations it generates [25].

The FEP and psychoanalysis are systemic theories. Attempts to embody and encapsulate free energy—through practice, gesture, speech, or, indeed, silence—are constrained by the need to tread a narrow path between complexity and accuracy. Ex cathedra analytic ‘interpretation’ may be a life raft for floundering psychoanalytic patients, but it often lacks veridicality. Likewise, in the world of free energy minimisation, there is always superfluity, energy that remains confoundingly unbound. Our models of the world can never fully comprehend its full complexity. The acknowledgement of this uncertainty underlies a particular strand of psychoanalytic thought which champions ‘negative capability’, associated with authors such as Bion [26], Ferro [27], Ogden [28], and Holmes and Storr [29]. We cannot fully comprehend the world in which we find ourselves, yet we cannot not try.

The phrase ‘negative capability’ (NC) was coined by John Keats [30] in a letter to his brother George, defining it as ‘*when a man is capable of being in uncertainties, mysteries, doubts, without any irritable reaching after fact and reason*’. For Keats, Shakespeare is NC’s foremost exponent, with his capacity to efface his own viewpoint and bring to life the conflicting personalities of his plays’ characters without ‘taking sides’. Both of these features are clearly relevant to the work of psychotherapists, who, alongside other skills, need to learn temporarily to put their own personality to one side and find ways to identify with the client’s worldview. For the purposes of my argument, a key point is that Keats formulated NC in the context of *dialogue*—with his brother, and by invoking a great dramatist. This takes us to Friston’s idea of ‘duets for one’ and how it relates to the work of Donald Winnicott.

### 3.2. Winnicott and Transitional Space

The psychoanalyst Donald Winnicott [31] coined the phrase ‘transitional object’ to describe the toys, handkerchiefs, and other possessions which many small children acquire and from which they are temporarily inseparable. Winnicott designated them as occupying a psychic zone that is neither self nor other, neither objective nor subjective, but occupying an ‘in between’ play space. Such objects are ‘transitional’ in that they occupy this intermediate zone between the child’s inner world of phantasy and the external world of reality. An inert object—a soft toy—is imbued with significance and meaning in the same way that we relate to the key people in our environment, but with one crucial difference: the transitional object is within the control—the pluripotential ‘omnipotence’—of the child and her/his phantasies. The child’s parents tend to validate this, seeing and protecting these objects as special playthings unique to that child’s individuality. 

From a FEM perspective, transitional objects can be used to develop priors and exercise active inference in ways that take account of the real world but which are not subject to the constraints of that world. A transitional object can be safely loved, hugged, hated, thrown away, recovered, and forgiven in ways which build up children’s store of interpersonal narratives that they need in order to negotiate their way through the contours of interpersonal life. 

Winnicott later extended the concept of transitionality:

‘transitional phenomena spread out over the whole intermediate territory between ‘inner psychic reality’ and ‘the external world *as perceived by two persons in common*’, that is to say, over the whole cultural field’.

([31] p. 6; my italics)

For children, transitional space is where they play. For adults, it is the zone of daydreams and imagination whose locus in neuroscience formulations is the Default Mode Network [32]. Arguably, *transitional space is always either actually or implicitly relational*. Another famous Winnicott trope is the idea of a child playing ‘alone in the presence of the mother’ [31]. Secure attachment provided by the caregiver enables children to explore this inner/outer world boundary. In adults, this relationality is internalized, but creativity, whether quotidian or cultural—poem, tune, score, brushstroke, knit-stich, carpenter’s bench, or chopped onion—is inherently *dialogic* in that there is an explicit or implicit other to or for whom it is addressed.

### 3.3. Relationality

Humans are hyper-social creatures. To survive, we need to be able to read each other’s minds. The evolutionary anthropologist Hrdy [33] sees the origins of this mind-reading in the collaborative child-rearing— ‘it takes a village to rear a child’—typical of our species. For her, evolutionary fitness depends not on speed or strength but our complex prefrontal cortex and, relatedly, the complexities of our relationships with our conspecifics. This links to the observation that, in the search for free energy minimisation, we recruit and rely on others, with whom we form, in Friston and Frith’s [13] phrase, ‘duets for one’. In the ‘duet for one’ envelope, the participants’ bottom-up, world-inputting, experience, and active inference overlap, thereby enhancing the adaptation and fitness of each. 

When it comes to reality modelling, as the cliché has it, two heads are generally better than one. But any head won’t do. Attachment relationships are specific (when a child is distressed, only Mum will do) and the same applies to the kinds of intimacy psychoanalysts attempt to generate with their clients. ‘Duets for one’ depend on our capacity to put ourselves in another’s shoes and to enter ‘we-mode’ thinking [24]. Secure attachment ensures biobehavioural synchrony or yoking between care seeker and caregiver [34], in contrast to strangers and acquaintances. Mother/infant dyads and romantic couples fulfil this criterion, and psychoanalysts aim for it vis-à-vis their clients. 

In the psychotherapeutic relationship, the negative capability of the therapist allows for a degree of safeguarded unpredictability; this, in turn, enables the embeddedness of the client’s priors to be relaxed and, through active inference, new top-down models to be generated and tested. For example, the focus of mentalization-based therapy (MBT) [23] is to help suicidal and relationally dysfunctional clients move from lone ‘I-mode’ and ‘me-mode’ thinking and feeling to ‘we-mode’ collaborative mentalizing. The therapist’s words and gestures and their clients’ parallel responses are the bridges that connect one brain to another. The client is encouraged to be more open to the granularity of incoming experience (‘free association’) and to relax simplistic and often self-defeating priors. By entering ‘we-mode’ with the therapist, the client makes new, more complex, and adaptive sense of her/his world. Just as the infant ‘borrows’ the caregiver’s brain to make sense of her/his experience, so too does the psychotherapy client draw on the therapist’s store of top-down narratives and capacity to face negativity and trauma without disgust, and thereby to minimize free energy in more complex and adaptive ways.

### 3.4. Transitionality and Psychotherapy

The typical psychotherapy patient is epistemically challenged [35]. Their transitional space is constricted. They don’t know whom or what to trust. Their trauma-formed models of the world trigger fear, avoidance, and confusion. They are either painfully pressed against an unyielding reality or trapped in the shadows of a tormented inner world. Epistemic foraging is inhibited. There is no thinking space between ‘is’ (the concrete reality of trauma) and ‘aught’ (wishes, hopes, and imaginings that things could have been and could be different).

Psychotherapy helps redress these limitations on transitionality in a number of ways. First, a zone of safety is created but where uncertainty is seen as legitimate, expectable, and faceable. Second, it is a shared, interpersonal holding zone where therapist and patient coexist, collaborate, and form a viable ‘duet for one’. This is yet another formulation of the therapeutic alliance, a powerful agent of therapeutic change in its own right, fostered by the empathic responsiveness of the therapist, whether gestural, verbal, or through touch [36]. 

Third, this zone is a play space neither wholly ‘real’ nor fully permeated with phantasy. Fourth, the therapist, on the basis of countertransference, makes conjectural interpretations in which the pain of uncertainty and the need for defences against it can, in the relative safety of the ‘we-mode’, be acknowledged. Fifth, it provides an imaginative workshop in which new models of the world, bypassing repression and thus better able to encompass clients’ bottom-up impressions, and thus to bind free energy, are co-created. Finally, it provides a top-down meta-view of oneself and oneself-in-relation-to-others, one’s strengths and limitations. This in turn enables the psychotherapy subject to engage with uncertainty, relax the embeddedness of priors, and so redress the free-energy paradox by helping the unknown to be accepted in all of its elusiveness.

Freud [37] was fascinated by ‘the uncanny’, by the bizarreness of dreams, and by the seemingly perverse and self-undermining workings of the unconscious. By contrast, the free energy perspective sees the workings of ‘the unconscious’ as adaptive: attempts, via avoidance, repression, splitting, and/or coercive interpersonal behaviours, however limiting or self-defeating these may prove to be in the longer term, to minimise free energy. The self–world discrepancies of psychosis are manifestations of energy binding gone awry. Freud [38] (1938) described paranoia or psychotic delusions as patches on a rent in the ego. FEP sees them as top-down attempts to minimise free energy, even at the expense of gross misapprehensions of reality [39]. Likewise, ‘dream work’ is not a manifestation of repression (disguised wishes) but mental ‘housekeeping’, sifting through the ‘day’s residues’, discarding the irrelevant, consolidating existing models of the world, and generating new stories ready to encompass as yet unmet dangers.

The Fristonian perspective enables a psychotherapy-defined zone to be described, incorporating the Winnicottian concept of transitional space. Its boundaries are defined: at one end is the internal world, teeming with memories, comforting and/or traumatic, and the established top-down priors with which we shape our worlds. At the other limit stands brute reality, internal and external, where we are pressed both against our inescapable physiology and the social and political ecology into which fate has arbitrarily thrown us. Therapeutic relatedness opens up the fluidity of the shared transitional space between these two bookends. Dreams, creativity, and imaginings point to new possibilities. Transitionality is where we live and where we feel most alive. Our psychic thriving and surviving depends on how we negotiate the space between these two poles and the energy as yet unbound that resides there. 

## 4. Conclusions: The Social Role of Psychotherapy

Winnicottian transitionality and ‘we-goism’ are in short supply. In our Western, individualised, secularised world. Collective ties have weakened. Unlike our hunter–gatherer forebears, we no longer share a common niche. Class, ethnicity, skin colour, wealth, education, migration, capital, appropriation, and consumerism confine us to our individual thought-boxes, silos, and echo-chambers. The gulfs between us—especially between the haves and the have-nots—is ever widening. All around us swirls psychic free energy demanding collective supra-organismal binding.

This need is, to an extent, met by our political and ethnic and religious affiliations and identifications. Here too, we seek help from many sources, including the new priesthood of counsellors and therapists. If lucky, we form a ‘duet for one’ with a therapist, individually or in a group. Because it is our free energy not theirs, our therapists can help us face, name, and trace the anachronisms of our pre-existing models and delineate the ways in which they lie at the root of our depression, anxiety, or psychosis. If things go well, we learn to develop more sophisticated working models with which to bind free energy.

The role of the metaphorically held hand of a therapist enables its subjects to make use of this transitional space. Free energy is jointly bound. Terror transforms into surprise, jitters into jouissance. Spontaneity supervenes over mere survival. New formulations of one’s life, one’s confusions, and one’s injuries—both inflicted and suffered—emerge. Eventually, the external shared space becomes internalised, no longer dependent on the continuing presence of the therapist. There is genuine healing, never completed perhaps, but no longer despaired of.

This perspective, with its built-in uncertainty, differentiates psychotherapy from life-coaching, management, and workplace supervision. To return to Keats [30] (p. 215), ‘we hate poetry that has a palpable design upon us’. The therapist has no explicit ‘agenda’, hidden or otherwise, other than mutually to explore with the client ‘what is going on’ and to give voice to the thoughts and feelings with which they struggle. Implicitly, however, there is an aim or goal, an ‘entelechy’—active inference leading to free energy minimising. Clients’ free energy prior to consulting room examination either threatens to overwhelm or is subject to rigid suppression. The therapist unearths this unbound energy and holds it up to the light so that it can be better bound and harnessed to drive new patterns of living. In this, the therapist adopts a position of ‘non-attachment’, not in the sense of avoidance or dismissal of affect, but as a hovering meta-level where the higher reaches in the hierarchy of free energy minimisation can be encountered and addressed.

The free energy principle holds that a fish or aquatic mammal *in* water is a mirror or imprint of its environment. By contrast, a fish *out* of water is flooded with free energy, a world for which its priors have no counterpart, sensory input for which it has no model or coherent behavioural response. Psychotherapy points to ways of living both with the human and non-human world that more closely align with their realties, and to a less entropic and more complexity-coherent future. But our times are out of joint; we are fish out of environmental equilibrium. Psychotherapy and its offshoots are part of a wider movement to help the beached whale of humanity back where it belongs and longs, often unawares, to return. Fristonian fundamentals provide a sound scientific underpinning for this urgent project.
